# Artificial neural network models: implementation of functional near-infrared spectroscopy-based spontaneous lie detection in an interactive scenario

**DOI:** 10.3389/fncom.2023.1286664

**Published:** 2024-01-24

**Authors:** M. Raheel Bhutta, Muhammad Umair Ali, Amad Zafar, Kwang Su Kim, Jong Hyuk Byun, Seung Won Lee

**Affiliations:** ^1^Department of Electrical and Computer Engineering, University of UTAH Asia Campus, Incheon, Republic of Korea; ^2^Department of Intelligent Mechatronics Engineering, Sejong University, Seoul, Republic of Korea; ^3^Department of Scientific Computing, Pukyong National University, Busan, Republic of Korea; ^4^Interdisciplinary Biology Laboratory (iBLab), Division of Biological Science, Graduate School of Science, Nagoya University, Nagoya, Japan; ^5^Department of Mathematics and Institute of Mathematical Science, Pusan National University, Busan, Republic of Korea; ^6^Finace Fishery Manufacture Industrial Mathematics Center on BigData, Pusan National University, Busan, Republic of Korea; ^7^Department of Precision Medicine, Sungkyunkwan University School of Medicine, Suwon, Republic of Korea

**Keywords:** spontaneous lie detection, deception, deep learning algorithm, functional near-infrared spectroscopy (fNIRS), classification

## Abstract

Deception is an inevitable occurrence in daily life. Various methods have been used to understand the mechanisms underlying brain deception. Moreover, numerous efforts have been undertaken to detect deception and truth-telling. Functional near-infrared spectroscopy (fNIRS) has great potential for neurological applications compared with other state-of-the-art methods. Therefore, an fNIRS-based spontaneous lie detection model was used in the present study. We interviewed 10 healthy subjects to identify deception using the fNIRS system. A card game frequently referred to as a bluff or cheat was introduced. This game was selected because its rules are ideal for testing our hypotheses. The optical probe of the fNIRS was placed on the subject’s forehead, and we acquired optical density signals, which were then converted into oxy-hemoglobin and deoxy-hemoglobin signals using the Modified Beer–Lambert law. The oxy-hemoglobin signal was preprocessed to eliminate noise. In this study, we proposed three artificial neural networks inspired by deep learning models, including AlexNet, ResNet, and GoogleNet, to classify deception and truth-telling. The proposed models achieved accuracies of 88.5%, 88.0%, and 90.0%, respectively. These proposed models were compared with other classification models, including k-nearest neighbor, linear support vector machines (SVM), quadratic SVM, cubic SVM, simple decision trees, and complex decision trees. These comparisons showed that the proposed models performed better than the other state-of-the-art methods.

## 1. Introduction

Deception is an intrinsic and unavoidable facet of our society, manifesting itself in everyday life. It is unsurprising for a person to encounter or be involved in multiple deceptive situations within a single day. Failure to identify deception has serious consequences for the victim. To avoid being deceived, people have begun to study the behavioral and physiological changes exhibited by deceivers. Hence, this study aimed to detect the differences between hemodynamic signals during spontaneous deception and classify between truth and lie during an interactive game paradigm.

In earlier times, people identified deceivers based on the deceiver’s personality or their own personal experiences (Freud and Strachey, 1962; Zuckerman et al., 1981b; Kleinmuntz and Szucko, 1984; Peterman and Anderson, 1999). Additionally, during earlier times, people often relied on myths based on religious norms to identify a person who was being untruthful (Trovillo, 1938). Advancements in scientific methods and new equipment, including polygraphs, have enabled us to better understand the cues of deception that are beyond the scope of religious beliefs, personal experience, and stereotypes (Brett et al., 1986; Varisai Mohamed et al., 2006). These physiological measures have revealed many new findings that provide the basis for numerous theories, such as the non-verbal leakage theory (Ekman et al., 1969), four-factor theory (Zuckerman et al., 1981a), and interpersonal deception theory (Buller and Burgoon, 1996). These theories have helped us understand why these cues of deception manifest in humans when attempting to deceive someone (Bond et al., 2014). Most of these theories agree that the intent and process of deception invoke changes in the deceiver’s behavior that result from changes in the person’s state of mind.

Many researchers have investigated different neurophysiological signals to identify changes in an individual’s mental state while they are attempting to deceive. One such technique is Electroencephalography (EEG), which records event-related potentials (ERPs) from the scalp of the brain (Abootalebi et al., 2009; Meijer et al., 2013). ERPs are mainly used to test knowledge of crime details that are only known to the criminals involved (Farwell and Donchin, 1991). This type of test is commonly known as the guilty knowledge test or concealed information test (Furedy and Ben-Shakhar, 1991; Elaad and BEN-SHAKHAR, 1997; Kong et al., 2012). EEG has excellent temporal resolution, enabling rapid detection of brain signals (Turnip et al., 2011; Chen et al., 2023), but exhibits poor spatial resolution, which cannot confine the brain area associated with the deception process.

Functional magnetic resonance imaging (fMRI) is another technique widely used to detect brain areas activated during deception. fMRI offers a substantial advantage in terms of high spatial resolution when compared to EEG (Spence et al., 2004). It can effectively localize changes in regional blood flow (Farah et al., 2014) and hence provides a comprehensive review of fMRI-based deception decoding. Because of the high cost of scanners and their bulky size, the use of fMRI is very limited in day-to-day human routines. Moreover, fMRI is highly sensitive to motion artifacts. Therefore, researchers have embarked on exploring an alternative brain imaging technique: functional near-infrared spectroscopy (fNIRS).

Using fNIRS, brain activity is measured through hemodynamic responses associated with neuronal behavior (Kamran and Hong, 2013; Santosa et al., 2013; Khan et al., 2014; Ruotsalo et al., 2023). The fNIRS can provide topographic (Obrig and Villringer, 2003; Wolf et al., 2007; Hu et al., 2011; Li et al., 2018) and tomographic brain images (Bluestone et al., 2001; Boas et al., 2004). Oxy-hemoglobin (HbO), deoxy-hemoglobin (HbR), and water are significant light absorbers, whereas skin, tissue, and bone are mainly transparent to near-infrared light within an optical window of 650–1,000 nm. Compared with EEG and fMRI, fNIRS offers a superior tradeoff between temporal and spatial resolutions. In one study (Irani et al., 2007) compared the features of fNIRS and fMRI and reported that fNIRS has excellent potential for psychotic and neurological applications because of its portability, simplicity, and insensitivity to motion artifacts compared to fMRI. fNIRS also has several advantages over other brain imaging techniques; it can be designed in a compact and portable form, is very cost-effective (Muehlemann et al., 2008; Bhutta et al., 2014; Toglia et al., 2022), and can be used in diverse fields such as neuroscience, brain-computer interfaces (Naseer and Hong, 2013a,b), and rehabilitation.

## 2. Literature review

Limited research has been conducted in the field of fNIRS-based deception decoding (Tian et al., 2009; Hu et al., 2012; Ding et al., 2013, 2014; Bhutta et al., 2015; Emberson et al., 2017; Quaresima and Ferrari, 2019). To detect deception, one study (Hu et al., 2012) employed a mock crime paradigm. Because individuals were instructed to provide deceptive or truthful responses at specified times and locations, this research, which was based on the concealed information test, did not incorporate a spontaneous paradigm. The first study to use fNIRS to identify the neural correlates of spontaneous deception was conducted by Ding et al. (2013). These aforementioned studies on fNIRS-based deception decoding have exclusively investigated cases of deceptions where the perpetrator lies to an unsuspecting victim; this type of deception occurs more frequently in casual social interactions. In contrast, there are also situations in which the perpetrator deliberately misleads the victim, even though both parties are fully aware of the attempt at deception. This type of circumstance is typically referred to as reverse psychology, and it frequently occurs in highly competitive settings, such as diplomatic meetings, political debates and elections, sports, card games (including gambling), and other various scenarios. In this scenario, the individual employing reverse psychology can deceive the victim not only by uttering a false statement but also by making a truthful remark. The deceiver may choose to speak the truth, knowing that the victim is aware of the deceptive intention, yet the victim interprets it as a lie, thus believing the contrary. Consequently, speaking the truth serves the deceiver’s purpose of misleading the victim.

Deep learning classifiers have been widely used recently. A deep neural network (DNN) is composed of multiple layers of nonlinear processing modules called neurons (Schmidhuber, 2015; Huve et al., 2018). These fully connected or semi-connected neurons receive inputs from previous consecutive neurons. DNN can achieve superior classification performance in comparison to linear classifiers, such as linear discernment analysis (LDA), support vector machine (SVM), and others when applied to signals (language and speech processing) or images (Collobert and Weston, 2008; Krizhevsky et al., 2012; Bianchini and Scarselli, 2014; Simonyan and Zisserman, 2014). Hence, DNN classifiers are also gaining attention in the biomedical field (Hudson and Cohen, 2000; Cireşan et al., 2013; Ronneberger et al., 2015).

Only a few studies have employed DNN for classification. Abibullaev et al. (2011) investigated the performance of a DNN in a four-class classification experiment and reported a maximum accuracy of 94%. Yi et al. (2013) used a DNN to classify left and right motor imagery with an average classification accuracy of 84%. Hennrich et al. (2015) reported a similar classification performance of DNN compared to that of other classifiers (such as LDA and SVM) in a three-mental task experiment. To the best of our knowledge, no previous study has used a DNN for spontaneous deception decoding using fNIRS.

In this study, we hypothesized that, in the real world, a deceiver can deceive another person not only by telling a lie but also by telling the truth. Therefore, the objectives of this study were to:

- compare the differences between the hemodynamic responses produced by spontaneous lying and stating the truth,
- classify between the lie and truth for an interactive game paradigm,
- develop three deep ANN models for classifying responses, and
- compare the performance of the proposed deep ANN with other classifiers, such as LDA and SVM, in a spontaneous deception decoding paradigm.

According to these findings, the fNIRS system can accurately identify changes in HbO signals during spontaneous lies and truths.

## 3. Materials and methods

### 3.1. Subjects

**Figure 1:**
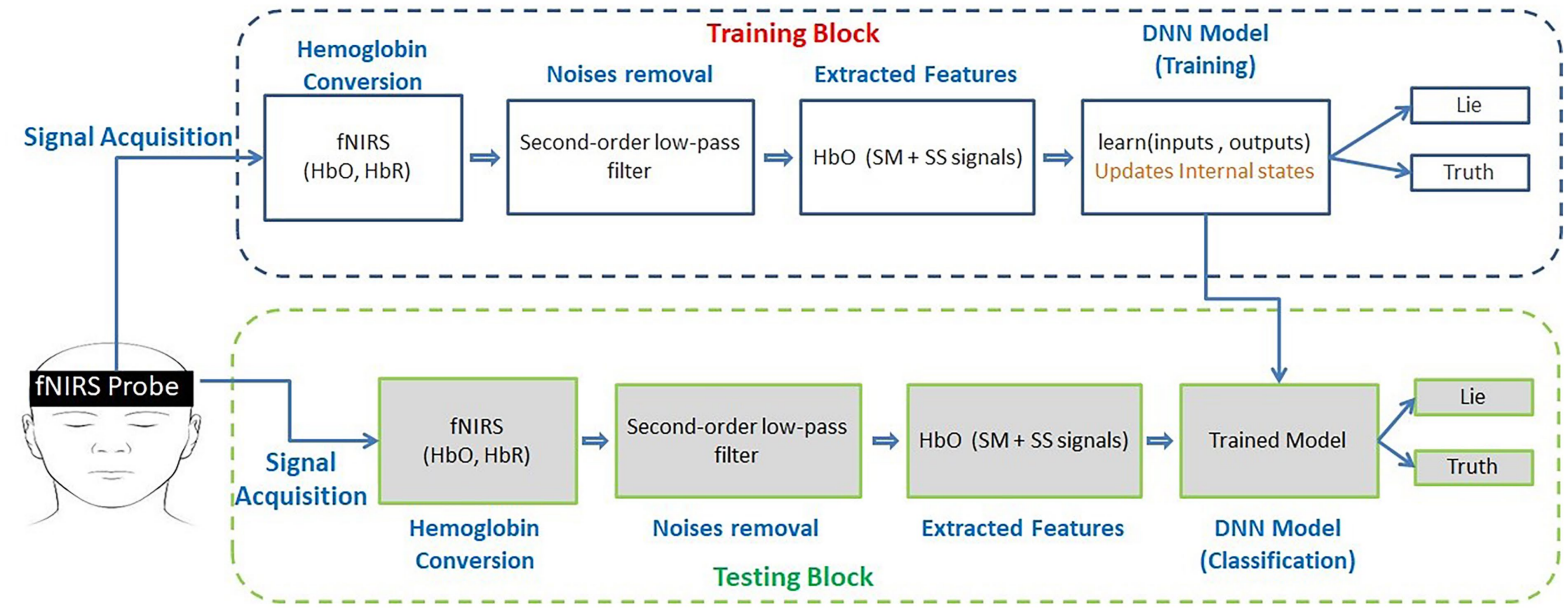
Proposed framework for spontaneous lie detection in an interactive scenario. SM: signal mean and SS: signal slope.

Ten healthy male individuals (mean age 30.8 ± 3.68) participated in the experiments. Each patient had normal or corrected-to-normal eyesight. Of the 10 subjects, nine were right-handed. None of the subjects had any history of mental or neurological illness. The card game was known to all subjects. Informed consent was obtained from all subjects, and the experiments were performed in accordance with the latest Declaration of Helsinki. The framework proposed in this study is illustrated in Figure 1. The framework is divided into two blocks: a training block (blue dotted lines) and a testing block (green dotted lines). The training black was used to train the neural network models on the given data, whereas the testing block was used to classify the data into truth and lie classes based on the model trained in the training block. Information on the individual blocks is provided in the respective chapters of the article.

### 3.2. Experimental procedure

The subjects were seated comfortably in front of a second person (referred to as the victim). The subject and victims underwent three practice sessions, and a brief explanation of the experiment before the experiment was provided to ensure that they fully understood the guidelines.

A well-selected experimental paradigm was used in this study. The experimental paradigm was a card game known as bluff or cheat. The bluff game was chosen because the rules of the game are ideal for testing our hypotheses. Our objective was to distinguish between deceptive actions when the subject is speaking the truth and when they are intentionally deceiving the victim with a falsehood.

The game rules are straightforward. The subject received 20 randomly selected cards, with the consideration that a minimum of six of these cards had no corresponding matches. Therefore, the subject had to lie at least four or five times in order to get rid of those cards. The subject had to play out all of their cards without revealing any signs of bluffing. The subjects had 1 min to carefully organize all their cards prior to starting the experiment. The duration of each experiment was approximately 10 min, with each experiment having a maximum of 20 sessions, each lasting approximately 30 s. In each session, the first 15 s were allotted for card arrangement. The subject had to lie to the victim in the next 5 s (called “claim time”) by laying his cards face down on the table and declaring what kind of cards they are (for instance, “three sevens”). Depending on his claim, the subject could select any number of cards between two and four. However, this assertion may or may not be correct. The victim then had 10 s to react to the subject’s assertion (response time). If the victim believed that the subject is telling the truth, they could choose to pass, removing the pile from the table. However, if the victim suspected that the subject had lied in their claim, they had the option to flip the cards face up. If the subject lied, the pile was returned to the subject. However, if the subject was truthful, the pile was removed from the table, and the next session commenced. The game continued for 20 sessions. A prize of 10,000 Korean Won was to be awarded to the subject if they managed to play all their cards within 20 sessions; however, if they were to do so, they would not receive the prize money. There were 12 total subjects in this trial. Two respondents’ data were excluded from the analysis as they consistently spoke the truth at the beginning of trials and only lied towards the end, rendering their responses predictable. Eight out of ten subjects were able to play all of their cards. One administrator continuously monitored the experiment and documented the trials in which the subject deceived the victim.

### 3.3. Data acquisition

**Figure 2:**
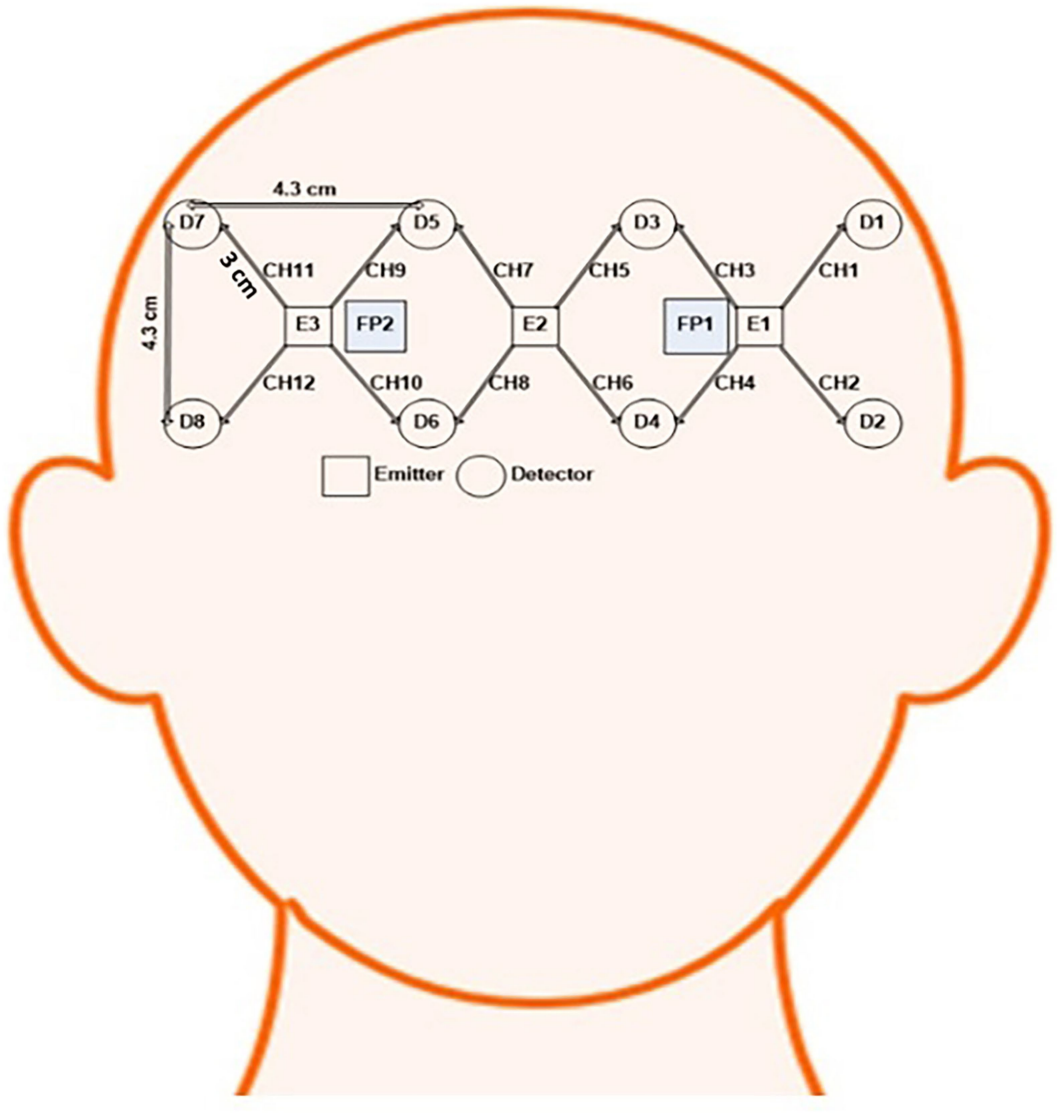
Optode placement and channel configuration.

A lab-built multichannel continuous-wave imaging system captured the brain signals (Bhutta and Hong, 2013). The optical probe of the fNIRS system was positioned on the forehead of the subject such that the FP1 and FP2 locations were in the middle of the probe, as shown in Figure 2. To connect the flexible probe and ensure excellent contact between the its emitters and detectors and the subject’s scalp, hair was brushed backward. Self-adhesive bandages were used to secure the probe to the subject’s head. The emitters and detectors were systematically positioned within a 4.3 × 13 cm^2^ area according to a source-detector distance of approximately 3 cm. A sampling rate of approximately 3.8 Hz was used to collect the data. A Velcro band was used to hold the probe at the appropriate location throughout the experiment.

### 3.4. Data processing

MATLAB (MathWorks, United States) was used to import and further analyze the signals from the fNIRS equipment offline. The data were stored in a host computer text file as digitized raw intensity values from the fNIRS system. The hemoglobin conversion block of the framework was used to convert the intensity values to concentration changes of HbO and HbR using the Modified Beer–Lambert law (Bhutta et al., 2015). The change in optical density (ΔOD) was calculated using these raw intensity values at each discrete time k as:


(1) $$\dot{\Delta OD\left(k;\lambda \right)}=\ln\frac{I_{\mathrm{out}\left(\lambda \right)}}{I_{\mathrm{in}}\left(k;\lambda \right)}=ld\left(\lambda \right)\Delta \mu_{a}\left(k;\lambda \right)$$

Where *I_out_* is the intensity of detected light; *I_in_*, intensity of incident light; *d*, differential path length factor; *l*, distance between the emitter and detector; and Δ*μ_a_*, absorption change of the tissue. The changes of HbO (Δ*c*_HbO_) and HbR (Δ*c*_HbR_) were measured using the modified Beer–Lambert Law as (Bhutta et al., 2015):


(2) $$\left[\begin{array}{cc} \Delta c_{HbO} & \left(k\right)\\ \Delta c_{HbR} & \left(k\right) \end{array}\right]={\left[\begin{array}{cc} ld^{\lambda_{1}\alpha_{Hbo}^{\lambda_{1}}} & ld^{\lambda_{1}\alpha_{HbR}^{\lambda_{1}}}\\ ld^{\lambda_{2}\alpha_{Hbo}^{\lambda_{2}}} & ld^{\lambda_{2}\alpha_{HbR}^{\lambda_{2}}} \end{array}\right]}^{-1}\times \left[\begin{array}{c} \Delta OD_{1}\left(k;\lambda \right)\\ \Delta OD_{2}\left(k;\lambda \right) \end{array}\right]\;$$

with λ_1_ = 640 nm, λ_2_ = 910 nm, *d*^λ^_1_ = 6.63, and *d*^λ^_2_ = 2.765, according to the values for the wavelength-dependent absorption coefficients *α*_HbO_, *α*_HbR_. fNIRS, while detecting the hemodynamic responses, picks up the physiological noise of respiration, pulse, and low-frequency drift fluctuations. A second-order low-pass filter with a cutoff frequency of 0.15 Hz was used to eliminate such noises (Hu et al., 2012; Bhutta et al., 2015). The HbO was considered for further analysis in this study because it is a more sensitive and reliable activity indicator than HbR (Hoshi, 2003, 2007).

### 3.5. Classification

Once the data were preprocessed, classification was performed on the Δ*c*_HbO_(*k*) signals. We conducted this classification to distinguish between lie and truth responses based on the features extracted from fNIRS signals. The features selected in this study were the signal mean (SM) and signal slope (SS) of the HbO signal during the 5-s claim period of the stimulus. We used this claim period because it is the actual time at which the subject attempted to deceive the victim by either telling the truth or lying. The average HbO signal for each subject was obtained by averaging all 12 channels of the corresponding subject. SM and SS values over a 5-s window can yield better results in binary classification (Khan et al., 2014; Bhutta et al., 2015).

In this study, we performed the classification using various classifiers categorized into linear and nonlinear categories. LDA and SVM are the main linear classifiers, whereas the ANN is a nonlinear classifier. Both the LDA and SVM algorithms classify different classes of data based on hyperplanes. In LDA, a separating hyperplane is generated to minimize the interclass variance and maximize the distance between the class means (Lotte et al., 2007). For the SVM classifier, a separating hyperplane is designed such that the distance between the hyperplane and the nearest training point(s) is maximized (Naseer et al., 2014).

Mainstream machine learning techniques can be categorized as linear or nonlinear classifiers. Linear classifiers classify a sample based on the value of the linear combination of its features. For example, assume that we have an input feature vector x. A linear classifier then constructs a function that directly assigns the input vector x to a specific class:


(3) $$\text{f}\left(\text{x}\right)=\;\;\;\{\begin{array}{c} 1\;\;\;\text{if}\;\text{x}>\text{threshold},\\ -1\;\;\;\text{otherwise} \end{array}$$

A linear SVM is a linear classifier that makes decisions according to a linear hyperplane capable of effectively segregating data. SVM finds an optimal hyperplane by maximizing the margin, which is the minimum distance between the hyperplane and any of the data samples. Such classifiers perform well when the problem is linearly separable. However, if the data are not linearly separable, they will have poor generalization ability. In this case, we could map the input vector onto a higher-dimensional space using the kernel function K and find the separating hyperplane in that particular dimension. Quadratic SVM and Cubic SVM are examples of kernelized versions of SVM that utilize second and third-degree polynomial kernels.


(4) $$\text{K}\left({\text{x}}_{\text{i}}{\text{,x}}_{\text{j}}\right)={\left({\text{x}}_{\text{i}}^{\text{T}}{\text{x}}_{\text{j}}+1\right)}^{\rho }$$

In the machine learning literature, several other algorithms handle nonlinear cases using a completely different computational approach; one of the simplest algorithms is the K-Nearest Neighbor (KNN). The main idea of this algorithm is that, for a new instance to be classified, the algorithm searches for the K-nearest points in the feature space and assigns it to the class that prevails among its neighbors. Similarly, the decision tree constructs a classification model with a tree-like structure. It partitions a feature space into smaller regions containing homogenous instances and simultaneously incrementally constructs an associated decision tree. The partitions of the feature space are usually based on criteria such as the Gini impurity, information gain, or distance measure.

### 3.6. Proposed artificial neural networks (ANN) models

**Figure 3:**
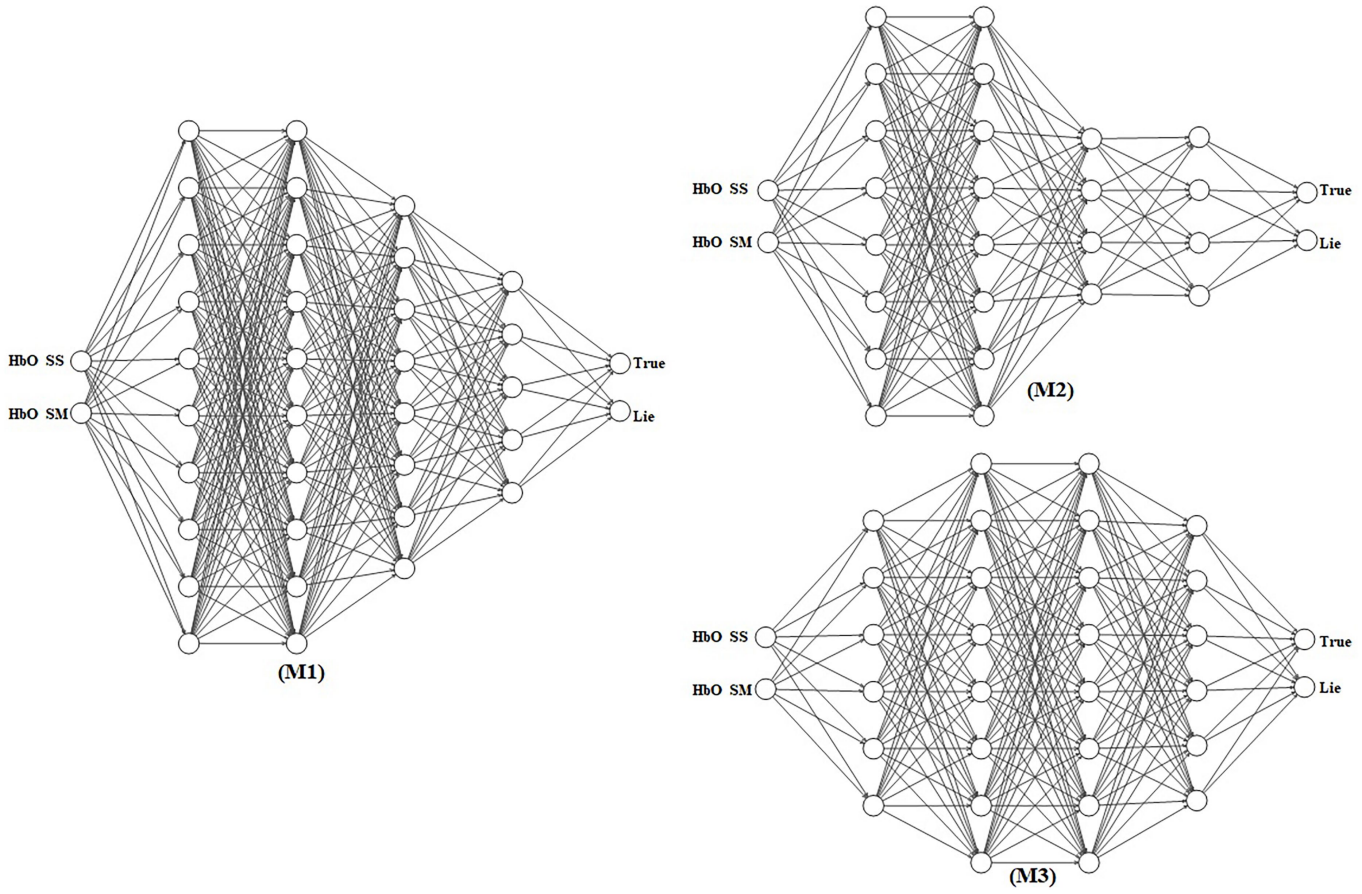
Neural network architectures for lie detection. Models M1, M2, and M3 process the mean and slope of the oxy-hemoglobin (HbO) signals through varying numbers of hidden layers and neurons. Each model produces a two-dimensional output representing the probabilities of a lie and truth.

In recent years, artificial neural networks have flourished in the machine learning and pattern recognition domains. They consist of many interconnected processing units, called neurons. The outputs of the hidden layer neurons are transmitted to the inputs of the next hidden layer within the network (Ullah et al., 2020). Thus, they communicate with each other by emitting signals over numerous weighted connections. During training, each neuron can update its weight, allowing the network to learn hidden patterns from the data. In this study, we designed three ANN architectures (M1, M2, and M3) to conduct experiments on our dataset. These structures were designed based on ideas from state-of-the-art convolutional neural network models, including AlexNet, ResNet, and GoogleNet. The numbers of input and output nodes and hidden layers of these neural networks are the same; however, the number of nodes in each hidden layer varies. The first two layers of M1 contain 10 neurons; the subsequent two hidden layers consist of eight and five neurons, respectively; and finally, the prediction layer contains two SoftMax classifiers. The M2 topology is similar to that of M1; however, we introduced two pairs of hidden layers with the same number of neurons in this structure. The first two layers had eight neurons, and the next pair had layers containing four neurons. We designed a third neural network architecture that differed from the aforementioned architecture. In this structure, we first increased the number of neuron dimensions from two to six and six to eight and then decreased it from eight to six and six to two neurons for the final class prediction. The architectures of the three ANNs are shown in Figure 3. Neural networks have weights that are initially randomly initialized, and later in the training process, these weights are optimized. The initial weights of our neural networks were determined using Kaiming uniform initialization (also known as HE initialization). This method is tailored for layers activated by the ReLU function and provides an advantage over random initialization. Specifically, HE initialization mitigates issues such as vanishing and exploding gradient problems, thereby enabling faster convergence during training. Aligning with the characteristics of ReLU, it also minimizes the occurrence of inactive neurons at the start of training. The empirical robustness of this method makes it a superior choice for deep network initialization compared to other simplistic methods. We selected four intermediate layers to achieve an optimal balance for our dataset. With only two features present in the input, it is essential to project them into a higher-dimensional space for feature extraction and subsequently condense the dimensions as we approach the classification layer. If we were to increase the number of hidden layers, the model would risk succumbing to the vanishing gradient problem. This is especially pertinent when processing only two features across excessive layers, as this is not advisable.

## 4. Results and discussion

This section presents a comparative analysis of the six statistical machine-learning techniques and three neural network models. The experiments were conducted using the MATLAB 2018b classification learning toolbox and Python 3.5 with Keras. We utilized a confusion matrix, receiver operating curve (ROC), area under the curve (AUC), and subject-level performance evaluation for the proposed method, which are discussed in subsequent sections.

In the domain of machine learning, mainly while dealing with classification problems having a distinction between a number of different items, the confusion matrix is considered an effective metric for evaluation. It is also known as the error matrix, as it indicates the error rate. It is used to show the effectiveness and performance of any trained classifier and summarizes the prediction results on any classification problem. We used a confusion matrix as an evaluation metric to demonstrate the performance of our proposed method.

**Figure 4:**
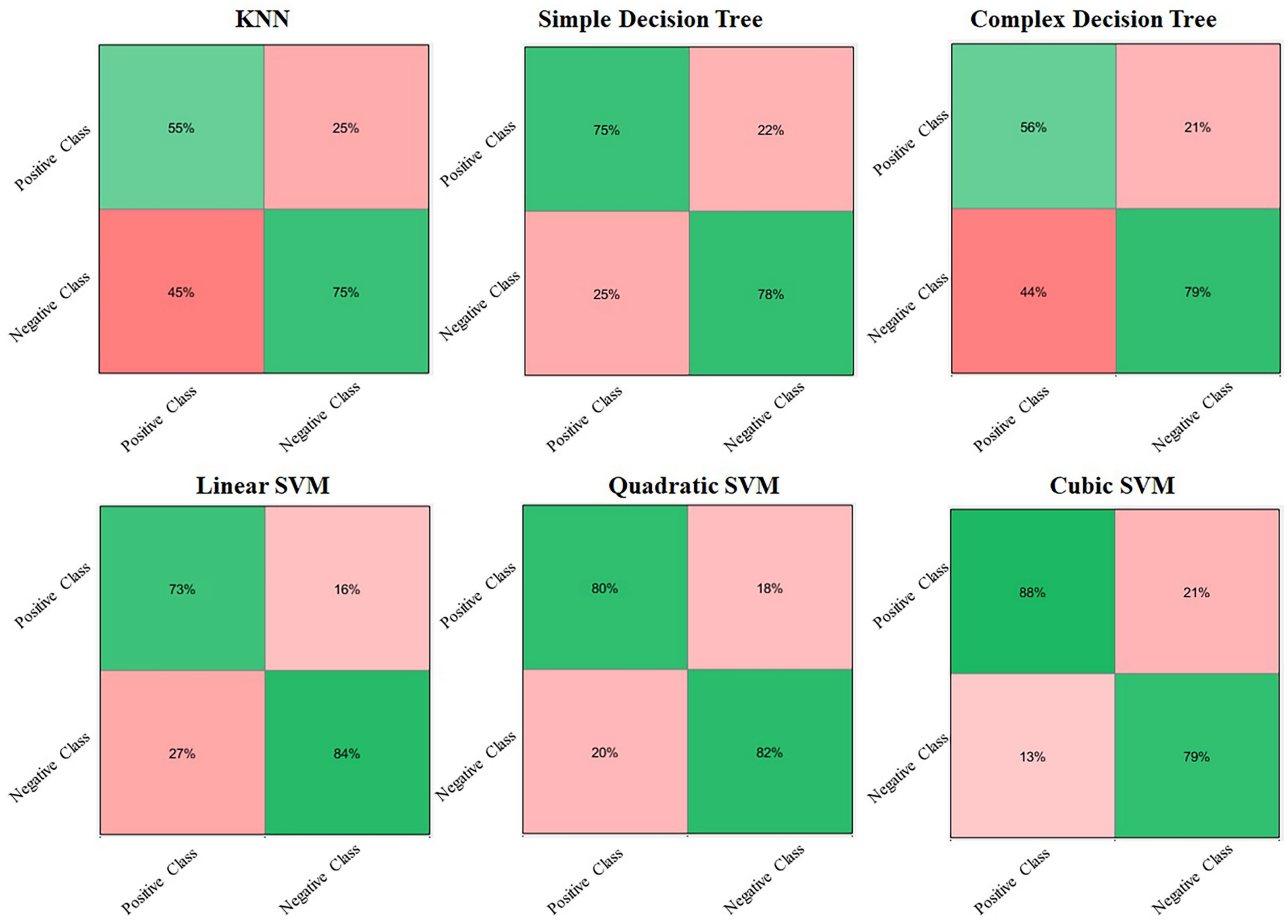
Confusion matrices of different statistical machine learning classifiers for lie prediction.

The predictive class-wise results for different classifiers with different statistical classifier flavors are shown in Figure 4. The top left corner in Figure 4 shows the confusion matrix for the KNN classifier, followed by simple and complex decision trees with 55%, 77%, and 56% completely true predictions, respectively. The accuracy achieved by these classifiers for positive classes is not convincing for real-world problems or for their deployment in different sectors. Therefore, we obtained better prediction results for the same data using different classifiers in the second row, starting from the linear SVM, followed by the quadratic and cubic SVM. The quadratic SVM achieved an average correct prediction result of 80%, which was dominated by the cubic SVM. The cubic SVM obtained the highest prediction results, with 88% correct prediction results for the positive class on the same data, proving it to be the best fit for deployment and practical implementation in real-world lie detection problems. The overall accuracy performances of different classifiers are listed in Table 1. Table 1 shows that the three proposed models were dominant for all statistical machine learning classifiers and achieved 8%–10% of the overall accuracy of the system.

**Table 1:** Comparison of different machine learning classifiers for overall accuracy.

Method	Overall accuracy (%)
KNN	68.5
Simple decision tree	77.5
Complex decision tree	70.0
Linear SVM	80.0
Quadratic SVM	81.5
Cubic SVM	80.5
Proposed NN M1	88.5
Proposed NN M2	88.0
Proposed NN M3	90.0

KNN, k-nearest neighbor; SVM, support vector machines; NN, neural network.

### 4.1. ROC and AUC curves

In a binary classification problem, the output class is usually labeled as positive or negative. The classification results can be represented in a structured form called a confusion matrix. However, the confusion matrix only provides true- and false-positive results. Therefore, to check the performance of the classification model at different thresholds, we calculated the ROC curves for all classifiers. This ROC curve plots the True Positive Rate (TPR) and False Positive Rate (FPR) at various thresholds, where TPR is a synonym for recall. These can be defined as follows:


(5) $$\text{TPR}=\frac{\text{TP}}{\text{TP}+\text{FN}}$$

(6) $$\text{FPR}=\frac{\text{FP}}{\text{FP}+\text{TN}}$$

**Figure 5:**
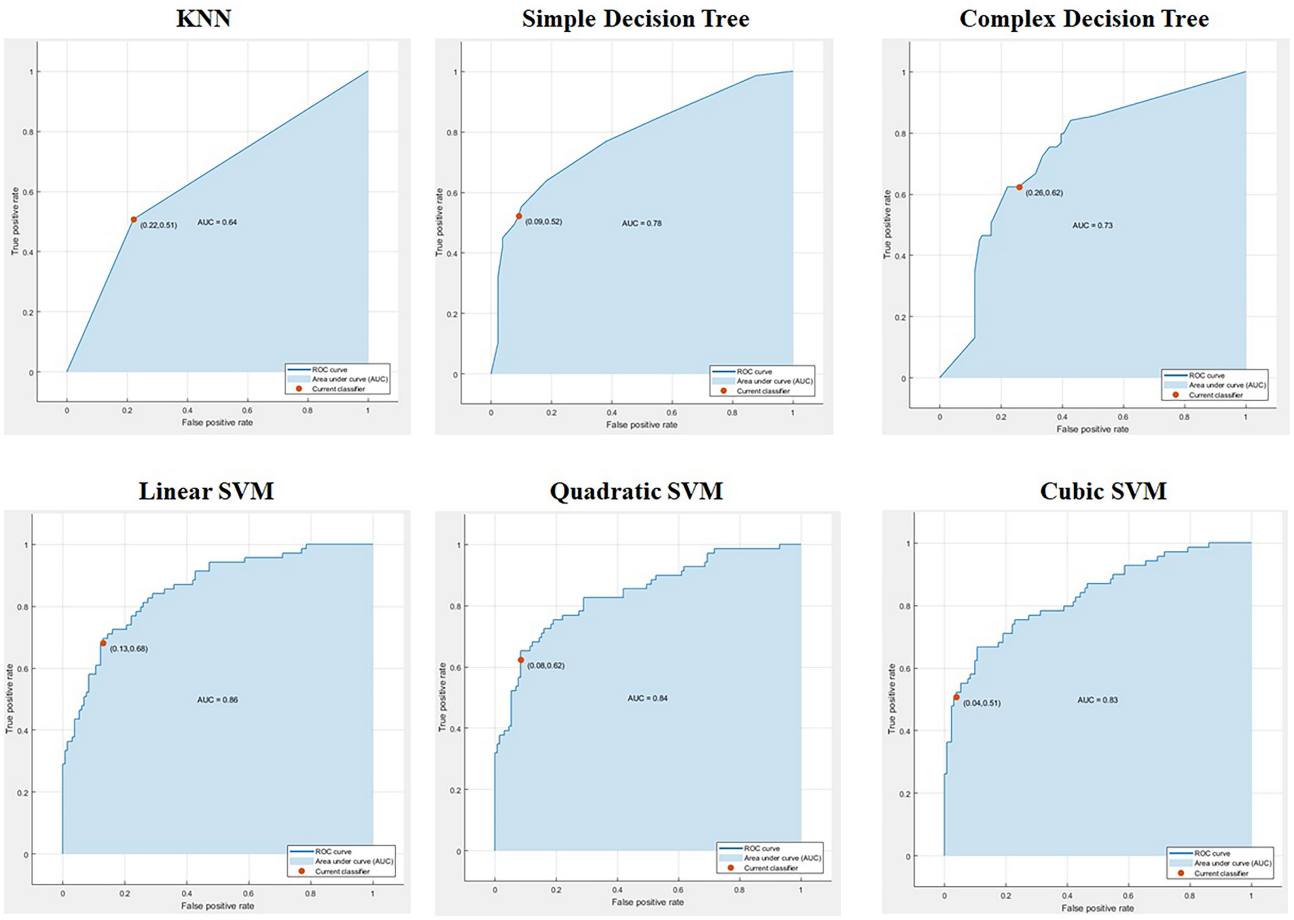
Receiver operating characteristic (ROC) curves and the area under the curve (AUC) values achieved from different hyperplane thresholds of six machine learning classifiers.

Moreover, for further evaluation, it is crucial to compute the ROC points, which is a resource-intensive method. An efficient sorting-based algorithm called the AUC, provides this information for evaluation. It measures the entire area under the ROC curve from (0,0) to (1,1). AUC offers an aggregate measure of performance at all possible thresholds. Thus, we calculated these values and obtained promising results for both the ROC curves and AUC values for all classifiers. The obtained AUC values and ROC curves for all classifiers are shown in Figure 5. The SVM classifiers achieved better AUC values and ROC curves, obtaining 86%, 84%, and 83% AUC for linear, quadratic, and cubic SVM, respectively. In contrast, the KNN, simple decision tree, and complex decision tree achieved AUCs of 64%, 78%, and 73%, respectively. Linear SVM has better accuracy than other statistical machine-learning techniques. However, it is still ineffective for sensitive problems, such as lie detection. To achieve better performance, we proposed three different neural network structures that increased the accuracy of lie detection from 8% to 10%.

### 4.2. Evaluation of the proposed ANN models

**Figure 6:**
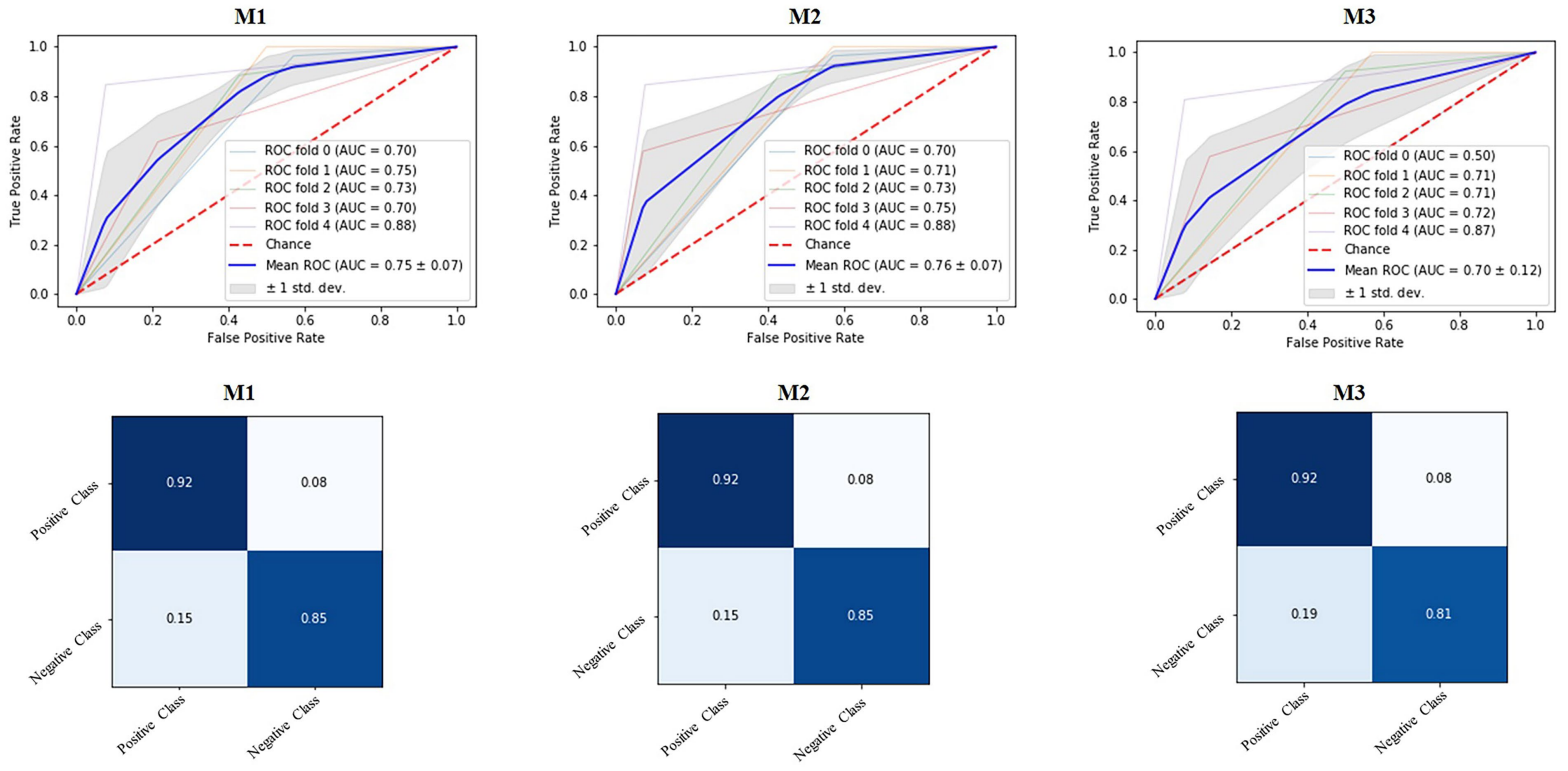
Receiver operating characteristic ROC curves, the area under the curve (AUC) values, and confusion matrices of three proposed neural network (NN) models for lie detection.

In the proposed method, we conducted experiments on our data using the three neural network models discussed in detail in the proposed methodology section. The models were trained for 50 epochs, and the data were divided into training, validation, and test sets of 60%, 20%, and 20%, respectively. The confusion matrices, ROC curves, and AUC for the three models are shown in Figure 6, and the overall accuracies are listed in Table 2. The proposed neural network models outperformed statistical machine learning approaches by a large margin, reaching 90% overall accuracy for the M3 neural network model, which is a 10% increase in accuracy. We trained our models five times and obtained the highest accuracies of 88%, 88%, and 87% for the fourth folds of M1, M2, and M3, respectively. The confusion matrices of the three models were almost identical, demonstrating the effectiveness of the models for lie detection.

**Table 2:** Results achieved by different trained models for sample test data.

Subject	Sample	Actual class	Prediction								
			NN M1	NN M2	NN M3	KNN	SDT	CDT	L-SVM	Q-SVM	C-SVM
1	1	True	True	True	True	True	True	True	True	True	True
	2	False	True	False	False	True	True	True	True	False	True
	3	True	True	True	True	True	True	True	True	True	True
2	1	False	True	True	True	True	True	False	True	True	True
	2	True	True	True	True	True	True	True	True	True	True
	3	False	False	True	False	True	False	True	True	True	True
3	1	True	True	True	True	True	True	True	True	True	True
	2	False	False	True	False	False	True	False	False	False	False
	3	True	True	True	True	True	True	True	True	True	True
4	1	False	False	False	False	False	False	False	False	False	False
	2	True	True	True	True	True	True	True	True	True	True
	3	False	True	True	True	True	True	True	False	True	True
5	1	True	True	True	True	True	True	True	True	True	True
	2	False	False	True	False	True	True	True	True	False	False
	3	True	True	True	True	True	True	True	True	True	True
6	1	False	False	False	False	False	False	False	False	False	False
	2	True	True	True	True	True	True	True	True	True	True
	3	False	True	True	True	True	True	True	True	True	True
7	1	True	True	True	True	True	True	False	True	True	True
	2	False	False	False	False	False	True	False	False	False	False
	3	True	True	True	True	False	True	False	False	False	False
8	1	False	False	False	False	False	False	False	False	False	False
	2	True	True	True	True	True	True	True	True	True	True
	3	False	True	False	False	True	True	True	True	True	True
9	1	True	True	True	True	True	True	True	True	True	True
	2	False	False	False	False	True	False	True	True	False	False
	3	True	True	True	True	False	True	False	False	False	False
10	1	False	False	False	False	False	False	False	False	False	False
	2	True	True	True	True	False	True	False	True	True	True
	3	False	True	False	False	True	True	True	False	False	False
Average accuracy (%)			80	80	90	60	70	60	70	77	73

KNN, k-nearest neighbor; SVM, support vector machines; NN, neural network; SDT, simple decision tree; CDT, complex decision tree; L-SVM, linear-SVM; Q-SVM, quadratic-SVM; C-SVM, cubic-SVM.

**Figure 7:**
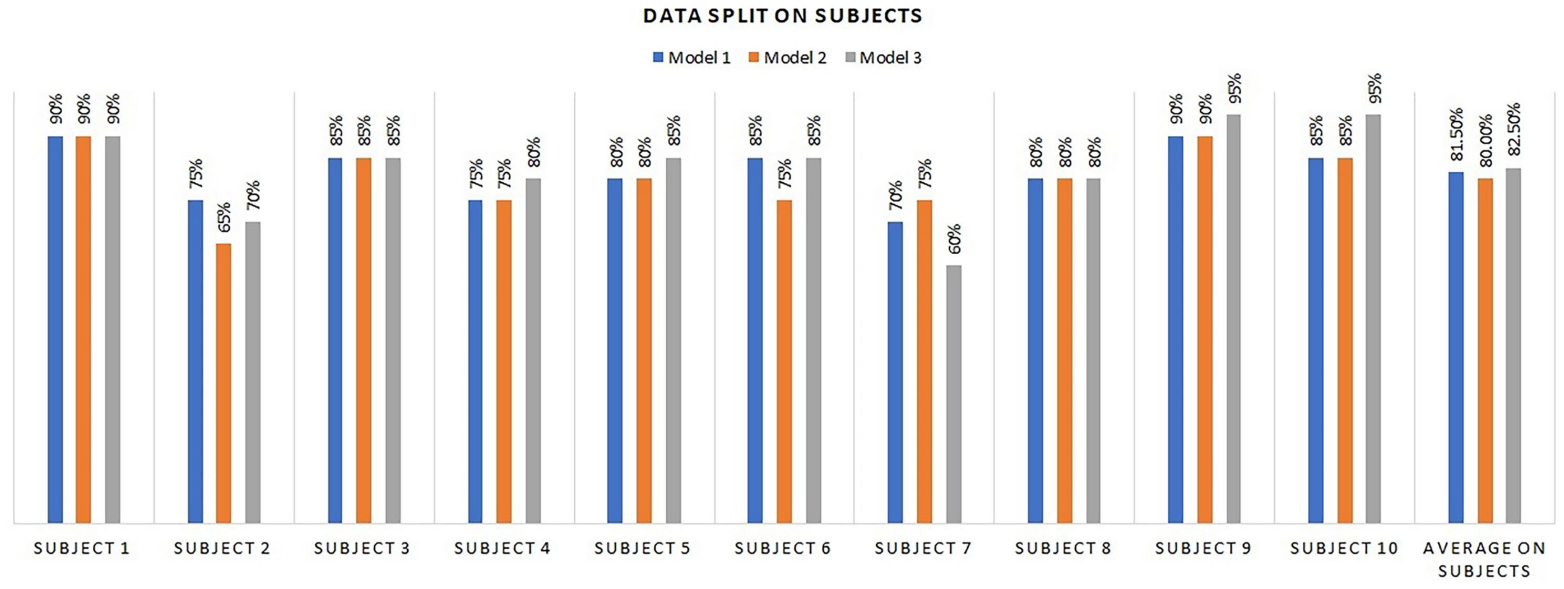
Subject-wise performance evaluation of the three proposed neural network (NN) models.

The proposed neural network models were also evaluated for subject-wise performance, which is illustrated in Figure 7. In the entire dataset, we had a total of 10 subjects. For this experiment, we trained our models on nine subjects and tested the models on the remaining one subject. This experiment showed the accuracy of our models when applied to unseen data. Subjects 1 and 9 achieved the highest accuracy of 90% and 95% on each model, respectively; only subjects 2 and 7 were had accuracies less than 70%. The remaining subjects had accuracies greater than 80% for all three models. The average accuracies achieved for M1, M2, and M3 were 81%, 80%, and 82%, respectively, demonstrating that the models are very effective and robust for unseen data.

Figure 7 displays the results for the test set of each subject’s data. We randomly selected three samples from each subject to check the robustness of our models for different subject’s data. The third column represents the actual label of the test sample, and the other columns represent the results of its corresponding machine-learning algorithm. The proposed three neural network models achieved better performance of 80%, 80%, and 90% subject-wise accuracy for neural network1, neural network2, and neural network3, respectively. On the other hand, the machine learning-based methods, namely KNN, SDT, CDT, L-SVM, Q-SVM, and C-SVM achieved 60%, 72%, 60%, 70%, 77%, and 73% accuracies, respectively. The proposed models have low accuracy for only three samples’ data, including the first sample of subject 2 and the third sample of subjects 4 and 6. However, for this data, other machine learning algorithms also faced challenges in detection. Subsequent examination of data revealed that these particular samples significantly differed from the rest of the dataset and exhibited substantial noise; therefore, the outcomes for these three samples were unsatisfactory.

## 5. Conclusion

In this study, we proposed an fNIRS-based spontaneous lie detection framework. The HbO and HbR signals were acquired using the fNIRS system. We used HbO SS and HbO SM as features in the classification of truths and lies. We developed an ANN, inspired by deep learning including AlexNet, ResNet, and GoogleNet, for classification during HbO concentration changes in an interactive environment. The proposed models, M1, M2, and M3, had overall accuracies of 88.5%, 88.0%, and 90.0%, respectively. We compared the results of the proposed ANN models with those of conventional classifiers such as KNN, simple decision tree, complex decision tree, linear SVM, quadratic SVM, and cubic SVM and found that the proposed ANN models outperformed conventional methods. In addition, we compared the individual subject accuracies and found higher accuracies for individual subjects. We further tested randomly selected samples from each subject, and the proposed ANN models M1, M2, and M3 achieved accuracies of 80%, 80%, and 90%, respectively. The resultant accuracies demonstrated the feasibility and robustness of practical fNIRS spontaneous lie detection in interactive scenarios.

## Data availability statement

The original contributions presented in the study are included in the article/supplementary material, further inquiries can be directed to the corresponding authors.

## Ethics statement

The experiments were performed in accordance with the latest Declaration of Helsinki. The study was conducted in accordance with the local legislation and institutional requirements. The participants provided their written informed consent to participate in this study.

## Author contributions

MB: Conceptualization, Methodology, Software, Writing – original draft. MA: Conceptualization, Methodology, Software, Writing – original draft. AZ: Formal analysis, Investigation, Visualization, Writing – review & editing, Validation. KK: Formal analysis, Writing – review & editing. JB: Funding acquisition, Project administration, Resources, Writing – review & editing. SL: Funding acquisition, Project administration, Resources, Supervision, Writing –review & editing.

## Glossary

**Table:** 

fNIRS	Functional near-infrared spectroscopy
SVM	Support vector machines
EEG	Electroencephalography
fMRI	Functional magnetic resonance imaging
HbO	Oxy-hemoglobin
HbR	Deoxy-hemoglobin
DNN	Deep neural network
LDA	Linear discernment analysis
SS	Signal slope
SM	Signal mean
KNN	K-nearest neighbor
ANN	Artificial neural networks
ROC	Receiver operating curve
AUC	Area under the curve
TPR	True Positive Rate
FPR	False Positive Rate
